# Scanned versus Fused-Reconstructed Oblique MR-Images for Assessment of the Tibiofibular Syndesmosis—Diagnostic PerFormance and Reader Agreement

**DOI:** 10.3390/diagnostics11020197

**Published:** 2021-01-29

**Authors:** Hannes Seuss, Matthias Hammon, Frank Roemer, Rafael Heiss, Rolf Janka, Michael Uder, Peter Dankerl

**Affiliations:** 1 Department of Radiology, University Hospital Erlangen, Maximiliansplatz 3, 91054 Erlangen, Germany; hannes.seuss@uk-erlangen.de (H.S.); matthias.hammon@gmail.com (M.H.); frank.roemer@uk-erlangen.de (F.R.); rafael.heiss@uk-erlangen.de (R.H.); rolf.janka@uk-erlangen.de (R.J.); michael.uder@uk-erlangen.de (M.U.); 2 Department of Radiology, Boston University School of Medicine, 820 Harrison Avenue, FGH Building, 3rd Floor, Boston, MA 02118, USA

**Keywords:** ankle MRI, syndesmosis injury, AITFL, image reconstruction, image fusion

## Abstract

To evaluate the diagnostic performance and reader agreement of a novel MRI image fusion method enabling the reconstruction of oblique images for the assessment of the tibiofibular syndesmosis. We evaluated 40 magnetic resonance imaging examinations of patients with ankle sprains (16 with ruptures and 24 without) for the presence of anteroinferior tibiofibular ligament rupture. For all patients, we performed a fusion of standard two-dimensional transversal and coronal 3 mm PDw TSE images into an oblique-fusion reconstruction (OFR) and compared these against conventionally scanned oblique sequence for the evaluation of the tibiofibular syndesmosis. To evaluate diagnostic performance, two expert readers independently read the OFR images twice. We analyzed sensitivity, specificity, negative and positive predictive values, accuracy, and agreement. Reader 1 misinterpreted one OFR as a false negative, demonstrating a sensitivity of 0.94 and specificity of 1.00, reader 2 demonstrated perfect accuracy. Intrareader agreement was almost perfect for reader 1 (α = 0.95) and was perfect for reader 2 (α = 1.00). Additionally, interreader agreement between all fusion sequence reads was almost perfect (α = 0.97). The proposed OFR enables reliable detection of anteroinferior tibiofibular ligament rupture with excellent inter- and intrareader agreement, making conventional scanning of oblique images redundant and supplies a method to retroactively create oblique images, e.g., from external examinations.

## 1. Introduction

Ankle sprains are the most common injury from athletic activity [1,2]. However, even more ankle joint injuries (50.7%) occur in everyday life, with an incidence of 2.15 per 1000 person/years [3], often leading to chronic symptoms including pain, weakness, and instability [4]. So-called high-ankle sprains that may include injury to the tibiofibular syndesmosis occur in up to one-quarter of all ankle sprains [5]. Of these, the syndesmosis is injured in roughly 10% of cases without an injury of the lateral ankle ligaments [6]. The tibiofibular syndesmosis consists of four separate ligaments [7], the anterior and posterior tibiofibular ligaments, the transverse tibiofibular ligament, and the interosseous ligament. The most affected ligament is the anteroinferior tibiofibular ligament (AITFL), whereas the involvement of the posteroinferior tibiofibular ligament commonly exhibits a pattern of bony avulsion [8]. Moreover, associated ankle injuries, for example, to the calcaneofibular ligament, are displayed more often in case of AITFL injury [9], potentially resulting in peroneal muscle atrophy [10] and ankle instability [11]. 

To reliably evaluate the tibiofibular syndesmosis, magnetic resonance imaging (MRI) is the method of choice [6]. For the dedicated evaluation of the AITFL and posteroinferior tibiofibular ligaments, the acquisition and analysis of oblique MRI scans have demonstrated usefulness [12,13]. Some authors have suggested that the evaluation of oblique images alone is superior for the diagnosis of syndesmosis ruptures as compared with standard evaluation protocols consisting of axial, coronal, and sagittal images [8]. The drawbacks of an additional acquisition of an oblique sequence include additional MRI scanning time and the need for standardized oblique angulation to optimally visualize the syndesmotic ligaments.

To solve these problems, we proposed and investigated a new reconstruction method utilizing image fusion to create oblique-fusion reconstruction (OFR) images and compare these to the conventionally scanned oblique images. For the image reconstruction, we utilized a commercially available three-dimensional (3D) tool utilizing the standard transversal and sagittal images acquired in a routine clinical examination protocol as suggested by the European Society of Skeletal Radiology (ESSR) [14] and the Workgroup Musculoskeletal Radiology of the German Radiological Society [15]. 

The aim of this study was to develop a simple, yet powerful postprocessing algorithm that enables radiologists to create oblique reconstructions of the tibiofibular syndesmosis without the need for acquiring additional sequences. We hypothesized that the fusion of multidimensional non-isotropic sequences acquired along the transversal and coronal planes (to oblique reformations) to be diagnostically beneficial and to compensate for the inherent loss of image quality. Further, we assessed the diagnostic performance of the proposed oblique-fusion reconstruction and hypothesis that it is sufficient for the diagnosis of AITFL injury. We compared the diagnostic performance of two expert musculoskeletal readers evaluating the OFR with the standard of reference, which is based on a consensus read of three different radiologists using all sequences and clinical information, including operative reports and follow-up data. We further compared reader agreement and evaluated the required time to perform the image reconstruction. If the OFR proves feasible and diagnostically sufficient this postprocessing algorithm promises multiple clinical implications. For instance, standard oblique images would not have to be physically acquired and MRI scanning time can be saved. Furthermore, OFR images can be reconstructed retrospectively, for instance, from external examinations in case the diagnosis is unclear or the surgeons would like the AITFL displayed properly.

## 2. Materials and Methods

### 2.1. Patient Characteristics

We included datasets from 40 consecutive patients who underwent a standard ankle MRI scan, including an additional acquired oblique sequence due to clinically suspected rupture of the syndesmosis. The mean age of the participants was 29.4 ± 16.5 years (range: 10–75 years), and the majority of patients were male (*n* = 29).

### 2.2. Scan Technique

All participants underwent the institution’s standard protocol ankle trauma MRI scan. The examination protocol followed the ESSR-suggested orientations and weightings and consisted of the following sequences:

- Proton-density-weighted (PDw) turbo spin echo (TSE) transversal with a matrix of 448 pixels (px) × 358 px, a repetition time (TR) of 334 ms; and an echo time (TE) of 65 ms.
- T1-weighted (T1w) TSE coronal with a matrix of 512 px × 512 px, a TR of 454 ms; and a TE of 11 ms.
- PDw TSE with fat suppression (fs) in coronal orientation with a matrix of 448 px × 358 px; a TR of 3310 ms; and a TE of 63 ms.
- PDw TSE with fs in sagittal orientation with a matrix of 512 px × 409 px; a TR of 2000 ms; and a TE of 40 ms.

All of these sequences were scanned with a 3-mm slice thickness and a maximum field-of-view [10]. 

Furthermore, an oblique PDw sequence was measured with a matrix of 448 px × 359 px; a TR of 1800 ms; a TE of 29 ms; a slice thickness of 2.5 mm; and a 45° angulation with respect to the tibial plafond [7,8,9]. Patients were scanned at a 1.5-T field strength (Magnetom Aera; Siemens Healthineers, Erlangen, Germany). We used dedicated 12‑channel ankle coils for signal reception.

### 2.3. Standard of Reference

Due to the experimental design of using consecutive patients, operative reports were only available for those patients who were diagnosed with a rupture in the original clinical report. Therefore, three experienced radiologists (H.S., M.H., R.J.) performed a consensus read utilizing all acquired sequences and clinical information, including physical examination protocols and follow-up data. During this review, they evaluated all patients for the presence of a ruptured AITFL. 

### 2.4. Reconstruction of Oblique-Fusion Reconstruction Images

**Figure 1:**
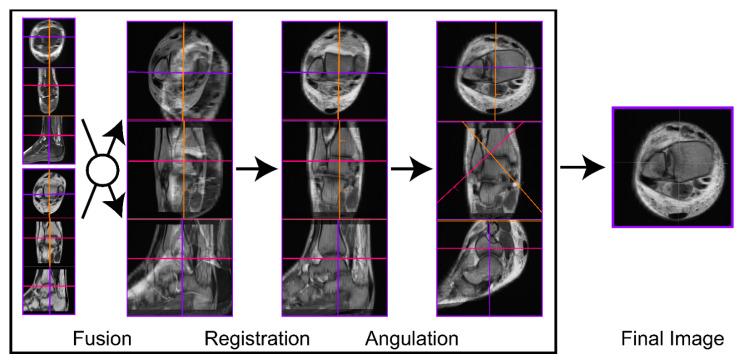
Oblique fusion reconstruction (OFR) was performed utilizing 3D postprocessing software. The transversal PDw and the sagittal PDw FS were first fused into one dataset, and then the two differently oriented image series were registered to create perfectly concordant images. From there, aligned in a 45° coronal angle to the tibial plafond, the oblique fusion images were reconstructed. The colored lines show the orientation of the displayed images in 3D.

Following the acquisition of the standard ankle MRI scans, the images were transferred to commercially available software (syngo.via, version VB20A; Siemens Healthineers, Erlangen, Germany). The scanned transversal PDw sequence and the sagittal PDw fs were loaded into syngo.via, where both sequences could be fused into a new dataset utilizing dedicated tools within the application. This is achieved first by creating an approximate overlay based on the spacing information in the Digital Imaging and Communications in Medicine header, while the fine-tuning is conducted via automated rigid registration with six degrees of freedom (3× translational and 3× rotational directions). From this fused dataset, oblique images were reconstructed with a slice thickness of 2 mm and in a 45° angulation with respect to the tibial plafond (Figure 1). Finally, these OFR images were archived in the picture archiving and communication system (PACS). The reconstruction of the OFR images was timed. Therefore, one research assistant performed the image fusion and reconstruction in all patients. 

### 2.5. Experimental Setup

We evaluated diagnostic performance by analyzing the accuracy and the reproducibility of the reads of two board-certified radiologists with 17 years and five years of (F.R., P.D.) experience in musculoskeletal radiology. They independently performed two separate experimental reads at least six weeks apart to avoid recall bias. They evaluated only the OFR images of all 40 patients while being blinded to the associated clinical information and in a random order. The two readers solely evaluated the AITFL and, within this experiment, were given the choice to diagnose the ligament as ruptured or intact. All Datasets were viewed on the same 30-inch screen with a resolution of 3280 px × 2048 px (Coronis Fusion 6MP LED (MDCC-6230); Barco, Kortrijk, Belgium). A study flow chart is available under Supplemental Material.

### 2.6. Statistical Analysis

Quantitative variables are expressed in the format of mean ± standard deviation, whereas categorical variables are expressed as frequencies. Sensitivity, specificity, positive predictive value (PPV), negative predictive value (NPV), and accuracy were calculated for every read using the consensus read as the reference. Differences in sensitivity and specificity were checked for significance using the chi-squared test. Intrareader agreement of the two OFR reads of one reader and interreader variability of the OFR reads of both readers (*n* = 4) were evaluated using Krippendorff’s alpha [16]. The Statistical Package for the Social Sciences (SPSS) version 21 software (IBM Corp., Armonk, NY, USA) was used for the analysis. A *p*-value of <0.05 was considered to be statistically significant.

The artwork was generated using SPSS version 21, Adobe Photoshop Extended, and Adobe Illustrator (both version CS6; Adobe Systems, San Jose, CA, USA).

## 3. Results

The reconstruction was completed within a few seconds. After the 3D workflow was loaded, it took on average 15 s (range: 12–19 s) to complete the fusion and to register the images. The reconstruction of the oblique images took 38 s (range: 29–48 s). The automated registration was successful in all cases.

**Figure 2:**
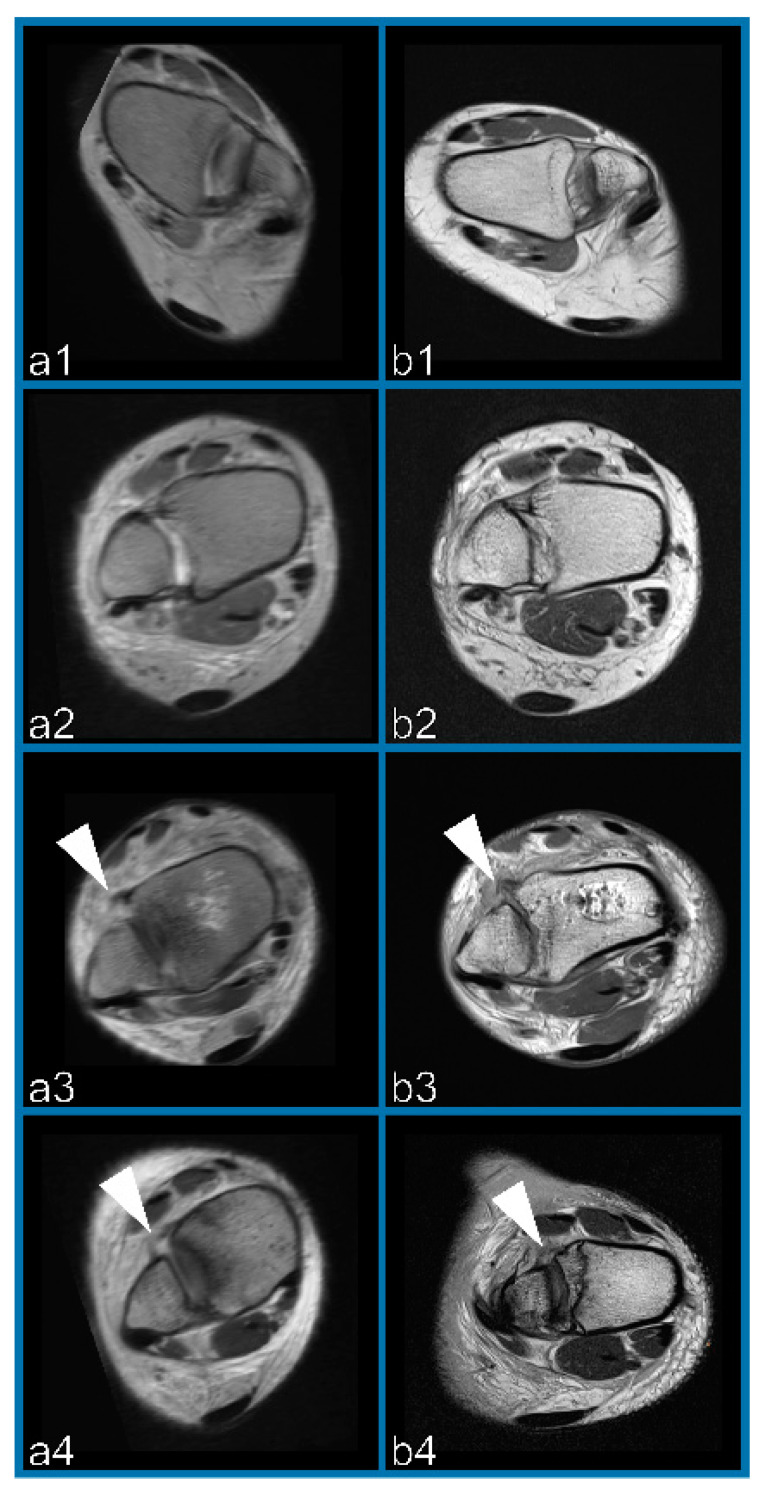
Example of two patients with intact (**1**,**2**) and ruptured (**3**,**4**) anteroinferior tibiofibular ligament (AITFL) in the OFR (**a**) and scanned (**b**) images. Arrowheads show the rupture.

In total, based on the consensus read, 16 patients presented with rupture of the AITFL and 24 presented without such, respectively (Figure 2). 

All 16 patients diagnosed with AITFL rupture underwent surgery, confirming a proven complete rupture of the AITFL. The 24 patients without MRI signs of AITFL rupture did not undergo surgery. 

During the first read, reader 1 was able to identify all patients correctly, whereas, in his second read, he missed one rupture (sensitivity: 0.94 (0.90–1.00)). Reader 2 evaluated all patients in both reads correctly. There were no significant differences between the two readers.

Intrareader agreement was almost perfect for reader 1 (α = 0.95) and was perfect for reader 2 (α = 1.00). Additionally, interreader agreement between all fusion sequence reads was almost perfect (α = 0.97). Detailed information is given in Table 1.

## 4. Discussion

From our results, we can show that the OFR images were not inferior to the measured oblique images of the AITFL. Intra- and interreader agreement were almost perfect.

Based on our findings, we propose that OFR images can replace physically scanned oblique MRI images for the evaluation of the tibiofibular syndesmosis in the setting of acute ankle sprain. As demonstrated, the OFR protocol provides diagnostically sufficient images without having to invest in MRI scanning time for an additional scanned sequence. The interreader evaluation demonstrated excellent agreement between readers. During their four reads, the two readers only achieved one false-negative rupture diagnosis with the use of the OFR protocol.

The OFR images are created from two differently oriented sequences (PDw sagittal and transversal). We state that this approach has the advantage of that the amount of spatial image information obtained by fusing two differently orientated sequences is higher than that obtained via reconstructing oblique images from just one sequence, thus drastically improving image quality and enabling the presented creation of OFR images of diagnostic quality.

As previously noted by Herrmans et al., the additional acquisition of 45° oblique images improves diagnostic performance in the examination of the tibiofibular syndesmosis, making these images desirable when evaluating ankle sprains [12,13].

Separately, Kim et al. suggested that an oblique sequence angulated in the same manner as our OFR is sufficient for the diagnosis of AITFL rupture [8]. The additional acquisition of the oblique PDw sequence used in their study required 3 min and 38 s of scanning time on average. In comparison, the additional oblique sequence commonly acquired in our institution required approximately 2 min and 24 s of scanning time—or 28% of the entire examination protocol. This scanning time would not be needed with the use of reconstructed OFR images. For the radiological reader to capitalize fully on the scan-time-saving ability of the OFR images, we proposed two solutions to incorporate the postprocessing that occurs into the workflow, as follows: either the reconstruction method is implemented as fully automatic into the software of MRI scanners, or the radiographer performing the MRI examination manually initiates the reconstruction on a separate 3D workstation and archives the OFR images as proposed by [17]. As presented, the manual creation of the OFR images took less than one minute and the step-by-step approach can easily be followed.

The two radiological readers who participated in the present study did not receive any training with the OFR images prior to completing their experimental reads, suggesting there is no need for additional training for the interpretation of the OFR images. 

**Figure 3:**
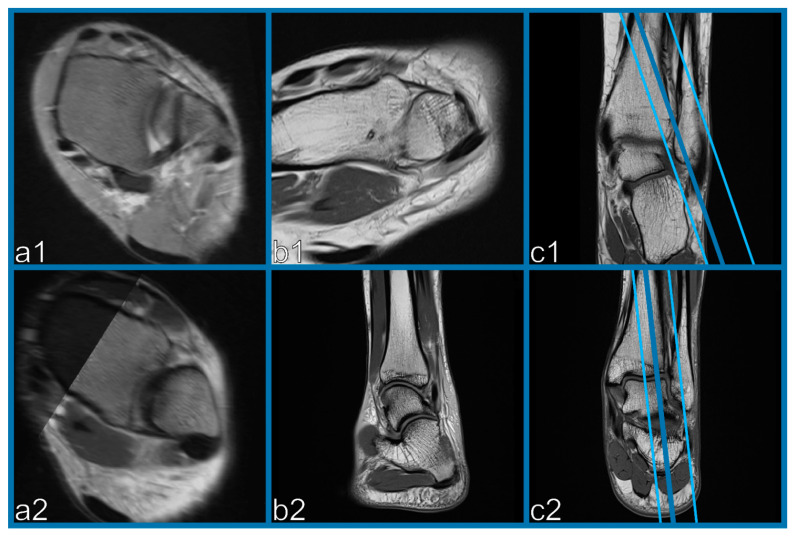
Example of two incorrectly scanned oblique sequences (**b1**,**b2**) with alignment angles greater than 60° (blue lines in **c1**,**c2**) with regards to the tibial plafond resulting in diagnostically insufficiently scanned oblique images (**b1**,**b2**). OFR images with correct angulation demonstrating one intact (**a1**) and one ruptured AITFL (**a2**).

In the clinical routine, image information concerning the integrity of the AITFL can be derived from standard axial, sagittal, and coronal images; however, these might not be conclusive in certain cases and the clinicians/surgeons may want to see the oblique images themselves. In some instances, we had to reexamine patients, as their scanned oblique images—as a result of errant angulation when planning the orientation—resulted in diagnostically insufficiently scanned oblique images (Figure 3). 

This problem can be solved with the proposed OFR protocol. Furthermore, in a time when, due to quality management constraints, preoperative patients have to be discussed interdisciplinarily in an image demonstration, and the clinicians/surgeons want to be presented with oblique images, the presented method enables the retroactive creation of OFR images of external examinations without scanned oblique sequences. 

As an alternative to the suggested image fusion using OFR, 3D isotropic TSE MRI sequences (e.g., SPACE, VISTA, or CUBE) can be used as well to investigate the AITFL. Nevertheless, this comes with some disadvantages. First, the substantially increased scan time seen with acquiring isotropic 3D sequences commonly does not provide substantial savings concerning overall scanning time as compared to two-dimensional (2D) sequences and has the disadvantage of being prone to motion artifacts. Moreover, the image quality of 3D sequences is still inferior to that of 2D images, as, when image noise is increased, image contrast may be decreased, and blurring is a common phenomenon [18]. Furthermore, in some health care systems (e.g., Germany), 3D sequences are not refunded.

Our study has some limitations that need mentioning. We did not have surgical information for the patients who were diagnosed with an intact syndesmosis, because these individuals did not undergo subsequent surgery. This might result in a degree of bias concerning sensitivity. However, all of the original clinical reports, the consensus read with the clinical information, the read of the whole examination, and the read of the acquired oblique sequence were in perfect concordance with one another. Further limitations include the small sample size and the retrospective nature of the study.

In future research, applying the presented method of fusing two differently oriented 2D sequences into a new dataset (e.g., oblique reconstructions of the cruciate ligaments or the peroneal tendons) could be performed.

## 5. Conclusions

The proposed postprocessing algorithm proved feasible and reliable to create OFR images in under one minute and demonstrated reliable detection of AITFL rupture with excellent inter- and intrareader agreement. 

This potentially makes the conventional scanning of oblique images redundant and supplies a method to retroactively create oblique images, e.g., from external examinations.

## Figures and Tables

**Table 1:** The diagnostic performances for the evaluation of the OFR images are shown for each reader. There were no significant differences between the OFR reads and the reference standard.

	Reader 1		Reader 2	
	1st Read	2nd Read	1st Read	2nd Read
TP	16	15	16	16
TN	24	24	24	24
FP	0	0	0	0
FN	0	1	0	0
Sens	1	0.94	1	1
Spec	1	1	1	1
PPV	1	1	1	1
NPV	1	0.96	1	1
Accuracy	1	0.98	1	1

TP = true positive, TN = true negative, FP = false positive, FN = false negative, Sens = sensitivity, Spec = specificity, PPV = positive predictive value, NPV = negative predictive value.

## Supplementary Materials

The following are available online at https://www.mdpi.com/2075-4418/11/2/197/s1.
