# Supplementary figures and images for: Scanned versus Fused-Reconstructed Oblique MR-Images for Assessment of the Tibiofibular Syndesmosis—Diagnostic PerFormance and Reader Agreement
Source: Diagnostics (Basel). 2021 Jan 29;11(2):197. doi: 10.3390/diagnostics11020197 (PMC7910886; doi:10.3390/diagnostics11020197)

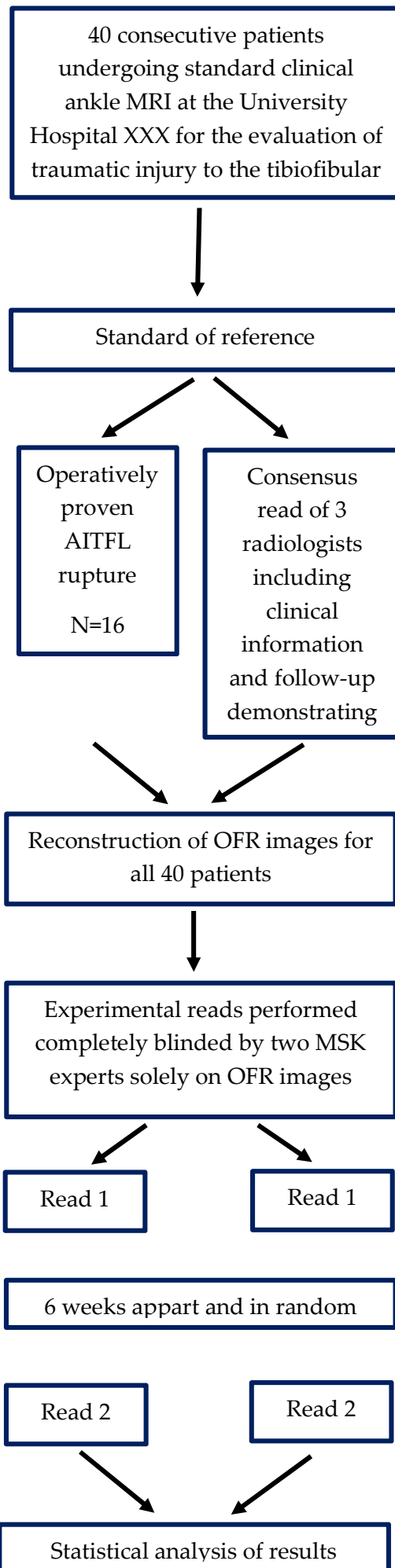

Supplement: Supplementary file 1 [file diagnostics-11-00197-s001.pdf]
